# The impact of microcrystalline and nanocrystalline cellulose on the antioxidant phenolic compounds level of the cultured *Artemisia absinthium*

**DOI:** 10.1038/s41598-023-50772-3

**Published:** 2024-02-01

**Authors:** Faezeh Khosravi, Samin Mohammadi, Morteza Kosari-Nasab, Parina Asgharian

**Affiliations:** 1 Student Research Committee, Faculty of Pharmacy, Tabriz University of Medical Sciences, Tabriz, Iran; 2 Department of Pharmacognosy, Faculty of Pharmacy, Tabriz University of Medical Sciences, Tabriz, Iran; 3 Department of Plant, Cell and Molecular Biology, Faculty of Natural Sciences, University of Tabriz, Tabriz, Iran; 4 Drug Applied Research Center, Tabriz University of Medical Sciences, Tabriz, Iran

**Keywords:** Plant sciences, Nanoscience and technology

## Abstract

*Artemisia absinthium* has long been used traditionally as an anti-microbial and antioxidant agent. Various biologically active secondary metabolites, including phenolic compounds such as gallic acid and p-coumaric acid, have been reported from the species. In addition, growing the plants under in vitro conditions enriched with elicitors is a cost-effective approach to enhance secondary metabolite production. This paper examined microcrystalline cellulose (MCC) and nanocrystalline cellulose (NCC) effects on morphological characteristics, phenolic compounds, antioxidant activity, and volatile oil content of *A. absinthium*. The treated shoots with various concentrations of MCC and NCC were subjected to spectrophotometric, GC–MS, and LC–MS analysis. FESEM-EDX, TEM, XRD, and DLS methods were applied to characterize MCC and NCC properties. Morphological findings revealed that the stem length, dry, and fresh weights were improved significantly (*P* ≤ 0.05) under several MCC and NCC concentrations. Some treatments enhanced gallic and *p*-coumaric acid levels in the plant. Although 1.5 g/L of MCC treatment showed the highest antioxidant activity, all NCC treatments reduced the antioxidant effect. The findings suggest that both MCC and NCC, at optimized concentrations, could be exploited as elicitors to improve the secondary metabolite production and morphological properties.

## Introduction

*Artemisia absinthium*, also known as wormwood, is a perennial woody plant belonging to the Asteraceae family. It has a bitter taste and a pleasant smell and is native to Europe. It grows in North Africa, Asia, South America, Canada, and the northern United States. The plant has been traditionally used for various medicinal purposes, such as being a bitter tonic, appetizer, disinfectant, vasodilator, anti-worm, anti-bacterial, anti-fungal, anti-viral, anti-parasite, antioxidant, anti-tumor, anti-spasmodic, choleretic, and diuretic^1–3^. *A. absinthium*, like many other medicinal plants, contains secondary metabolites that are Biologically active and responsible for the plant's medicinal properties. These secondary metabolites can be extracted and used as pharmaceuticals without modifications or transformed into a medicinal compound after undergoing semisynthetic processes^4,5^. Plants produce secondary metabolites that help them adapt to harsh environments. Some of the most important secondary metabolites in this plant are phenolic compounds like hydroxybenzoic acids (such as salicylic acid and gallic acid), hydroxycinnamic acids (like caffeic acid, coumaric acids, and ferulic acid), flavonoids (including myricetin, quercetin, rutin, and hesperidin), and volatile compounds (such as absinthin, artabsin, anabsinthin, and Matricin)^6^. However, producing these compounds takes a lot of work and money. To make it easier and cost-effective, traditional extraction methods have been replaced with growing the plant in a controlled environment using elicitors to induce the production of secondary metabolites in higher amounts.

Plant cell culture technologies were introduced in the late 1960s to study and produce secondary metabolites. Over the years, various techniques and elicitors have been widely investigated to improve secondary metabolite production^5,7^. One of the notable benefits of cell culture systems is their extensive scalability coupled with the capability of providing a sustained, reliable source of production and extraction of natural products^8^. Recently, cellulose-based structures have received great attention from researchers due to the increasing concerns about ecological issues. These components are the most abundant biopolymers in nature, and their physical, chemical, and mechanical stability, biocompatibility, renewability, non-toxicity or low toxicity, cheapness, availability, and unique surface properties have made them widely used in various fields, including industry, technology, and biomedicine. Microcrystalline cellulose (MCC) and nanocrystalline cellulose (NCC) belong to the various forms of cellulose based on their morphologies, performances, and sources^9–11^. Microcrystalline cellulose is produced commercially by acid degradation of cellulose fibers, which alters some of its amorphous regions. Its structure comprises interconnected nanocrystals with some amorphous regions^12^. Cellulose-based nanomaterials are classified into three groups: 1. Cellulose nanocrystals are mainly obtained from MCC and various natural fibers using the strong acid hydrolysis method. 2. Cellulose nanofibers are clusters of fibrils derived from sources like MCC and wood through mechanical procedures such as the homogenization process. 3. Bacterial nanocellulose, unlike the last classes, are formed via bottom-up technique from glucose. *Acetobacter xylinum* is one of the common species used^13^. The structure of nanocrystalline cellulose is made up of rigid rod-shaped particles that range from 5 to 30 nm in diameter and 100 to 500 nm in length^14,15^.

The application of microcrystalline cellulose and nanocrystalline cellulose in tissue culture has been limited to animal tissues like bone and skin tissue. There has been no documented case of using these materials in plant tissue culture. This study seeks to explore the effects of various concentrations of microcrystalline cellulose and nanocrystalline cellulose on the morphological characteristics, antioxidant activity, total phenolic and flavonoid contents, volatiles, and some polyphenolic compounds of *A. absinthium*.

## Materials and methods

### Provision of elicitors

Microcrystalline cellulose, sulfuric acid (H_2_SO_4_ 98%), and solvents were purchased from Merck (Germany), and nanocrystalline cellulose was obtained from microcrystalline cellulose using the acid hydrolysis method.

#### Preparation of Nanocrystalline cellulose

In a 100 mL round bottom flask equipped with a reflux condenser, 1 g microcrystalline cellulose was added gradually to 50 mL sulphuric acid (65% w/w). The reaction mixture was stirred moderately at 60 °C for 30 min. Subsequently, the mixture was poured into an ice bath and filtered by sintered glass to remove excess sulphuric acid. The sample was dispersed in water and centrifuged at 6000 rpm for 10 min. The process of washing was done several times to reach pH ~ 6.5. At last, the resulting NCC was dried under vacuum conditions for 18 h.

#### Characterization of NCC and MCC

The samples' surface morphology, particle size, and elemental composition were studied by field emission scanning electron microscope and energy dispersive X-ray (FESEM-EDX, MIRA 3 TESCAN company). X-ray Diffraction (XRD) was exerted to investigate the crystal structure of the samples (X' Pert Pro, Malvern Panalytical Ltd, Almelo, Netherlands). The TEM analysis was also carried out to assess the particle size and morphology of the samples by a CM120, Philips, Germany, operating at 200 kV. For TEM sample preparation, the sample was added to deionized water and dispersed ultrasonically using a probe-type ultrasonic generator (400 W). The samples' particle size distribution, polydispersity index (PDI), and zeta potential were characterized through dynamic light scattering (DLS). The sample for DLS was prepared in the same way as for TEM.

### Plant material

*Artemisia absinthium* *s*eeds were collected from the botanical garden of Tabriz University of Medical Sciences, situated in Tabriz, Iran (TBZFPH (No. 2283)). In order to sterilize the seeds, 70% ethanol and 20% sodium hypochlorite were used for 3 and 10 min, respectively, and at last, they were washed three times with sterile distilled water. The sterilized seeds were cultured in Murashige and Skoog (MS) medium with an adjusted pH of 5.6–5.8 and then kept under cool-white light illumination at 6480 lx and 25 ± 2 °C for 16 h/day. After about two months, the grown plants were used for explanting.

#### Organ cultures and treatments

The leaves and roots of each plant were discarded, and the stems were cut into 1–1.5 cm pieces so that each piece contained 2 or 3 nodes. Subsequently, they were explanted in untreated (as the control) and treated mediums with different concentrations of MCC (1.5, 3, 6 g/L) and NCC (1.5, 3, 6 g/L). After five weeks, growth parameters were measured per explant, including green and yellow leaf, node, root, seedling numbers, root and stem lengths, and fresh and dry weights. The air-dried plants were homogenized entirely, and a powder containing all parts of the plant (leaves, roots, and stems) was obtained. Two mg of the powder was soaked in 2 ml of 80% methanol. After 24 h in the dark, the extract was used to determine antioxidant activity and total phenolic and flavonoid contents.

#### Determination of the anti-oxidant activity (DPPH assay)

The antioxidant activity was measured using the DPPH method with slight modification^16^. An amount of 0.5 mL of methanolic extract was combined with 0.3 mL of 1 mM DPPH solution in 80% methanol. The final volume was increased to 3 mL with methanol. After 15 min incubation at room temperature, the absorbance was determined spectrophotometrically at 517 nm, and radical scavenging activity was calculated via the following equation.

$${\text{RSA}}\% = \left( {\frac{{{\text{Abs }}_{{{\text{DPPH}}}} - {\text{Abs }}_{{{\text{sample}}}} { }}}{{{\text{Abs}}_{{\text{ DPPH}}} }}} \right) \times 100$$

${{\text{Abs}}}_{{\text{DPPH}}}$ is the absorbance of the blank DPPH, and ${\mathrm{Abs }}_{{\text{sample}}}$ is the absorbance of the sample in the exposure of DPPH.

#### Determination of total phenolic content

The total phenolic content of the samples was assessed according to the Folin-Ciocalteu method^17^. An amount of 0.1 mL of the methanolic extract (1 mg/mL), 2.5 mL of distilled water, and 0.1 mL of Folin-Ciocalteu’s reagent were combined. After 6 min, 0.15 mL of 20% w/v sodium carbonate was added to the mixture, and after 30 min incubation at room temperature, the absorbance was determined spectrophotometrically at 760 nm. The results were expressed as mg equivalent of gallic acid per gram of dry weight.

#### Determination of total flavonoid content

The total flavonoid content of the samples was determined according to the aluminum chloride colorimetric method^18^. An amount of 0.5 mL of 2% w/v aluminum chloride was added to 0.5 mL of methanolic extract, and after 60 min incubation at room temperature, the absorbance was determined spectrophotometrically at 415 nm. The results were expressed as mg equivalent of quercetin per gram of dry weight.

#### Extraction of photosynthetic pigments

Wellburn’s method^19^ was used to determine the content of the photosynthetic pigments (chlorophylls a, b, and total carotenoids). Two mg of in vitro*-*produced shoots were extracted in 2 mL of dimethylformamide. The obtained extract was centrifuged (10,000 rpm for 10 min); eventually, the absorbance was determined spectrophotometrically at 480, 647, and 664 nm. The results were expressed as mg of pigments per g of fresh weight.

#### Extraction of volatile products

An amount of 77 mg of each sample was crushed and extracted using 5 mL of n-hexane for 15 min at room temperature. The final extract was filtered. One µL of the supernatant was subjected to GC–MS analysis to determine its volatile contents. Obtained extracts were kept at 4 °C^20^.

#### Gas chromatography-mass spectrometry (GC–MS)

Volatile compounds were undergone GC–MS analysis using a Shimadzu QP5050A with a DB-1 capillary column (60 m × 0.25 mm, 0.25 µm film thicknesses). The oven temperature program was as follows: The starting temperature was 60 °C, kept for 1 min, then increased to 290 °C at a rate of 8 °C/min and maintained at that temperature for 3 min. The injector's initial temperature was 270 °C, the split ratio was 1:10, and helium gas was applied as carrier gas at a 1.3 ml/min flow rate. Then, the components were identified by comparing the retention indices and mass spectra with previous article reports and the NIST library.

#### Extraction of polyphenolic compounds

One mg of the dried powder of each sample was extracted by 1 mL of 80% ethanol containing 100 µL of 2 M HCl to enhance phenolic compounds yield. After centrifuging, the final extracts were undergone LC–MS analysis to determine the phenolic acids profile^21^.

#### Liquid chromatography-mass spectrometry (LC–MS)

The grown shoots' phenolic acid profile was assessed through LC–MS analysis using a QUATTROZQ with a Thermo Hypersil-Keystone, Betasil C18 column (100 × 4 mm, 5 µm) at 35 °C. The phenolic acid standards were purchased from Merck Chemical Company. Mobile phases were water (A) and methanol (B), each having 0.1% formic acid. Multiple reaction monitoring (MRM) mode was applied for the detection of the following phenolic acids via monitoring the specified transitions: caffeic acid (178.9/134.9 m/z), gallic acid (169/125 m/z), ferulic acid (193/133.9 m/z), vanillic acid (167/152 m/z), m-coumaric acid (163/91 m/z), p-coumaric acid (162.9/118.9 m/z), salicylic acid (137/ 93 m/z), cinnamic acid (147/103 m/z), rosmarinic acid (359.1/197 m/z). The ESI was set in the negative mode, and the separation was done according to the timetable in Table 1. Subsequently, the phenolic acids were quantified according to the peak area compared to the standard curves. The results were expressed as mg of each compound per g of extract dry weight.

**Table 1:** The gradient timetable of the LC–MS analysis.

Time (min)	A (%)	B (%)	Flow rate (ml/min)
0	50	50	0.4
1	50	50	0.4
3	10	90	0.4
7	10	90	0.4
10	50	50	0.4

*A and B refer to methanol and water as mobile phases, respectively.

#### Sample preparation for scanning electron microscopy (SEM)

We examined air-dried shoots through scanning electron microscopy to study the morphological changes of the leaf and root surface. The images of MCC and NCC-treated shoots with a concentration of 1.5 g/L represented more changes than the control. Therefore, the pictures of the treatments mentioned above are given in the present manuscript.

#### Statistical analysis

All experiments were performed randomly with five replicates. The data were analyzed using analysis of variance (one-way ANOVA), and Duncan’s comparison means test with a significance level of *P* ≤ 0.05 in SPSS software version 16.0. In addition, all the methods used in the current study followed relevant institutional guidelines and regulations.

## Results and discussion

### Characterization of MCC and NCC

#### SEM

**Figure 1:**
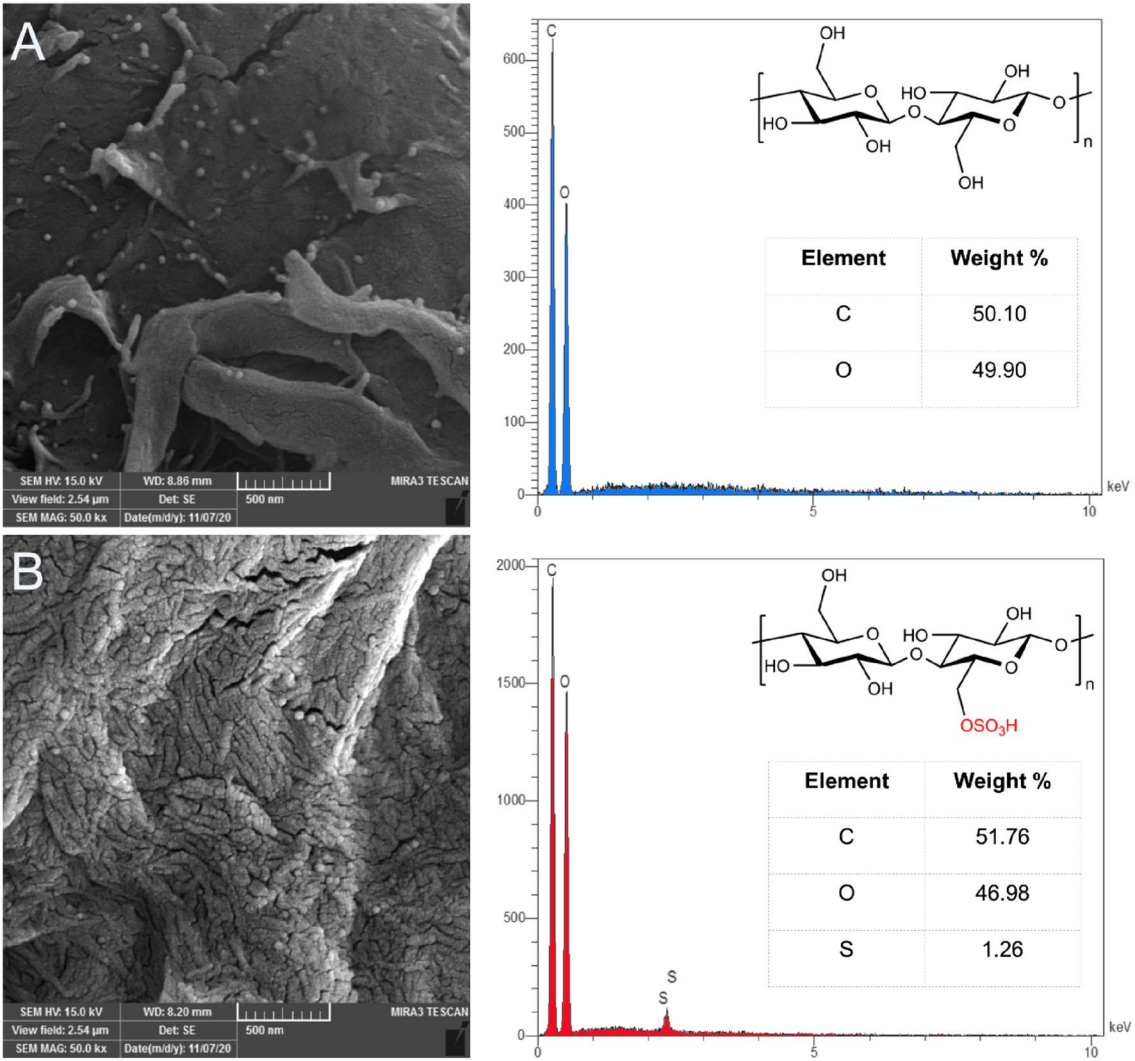
SEM and EDX analysis of MCC (**A**) and NCC (**B**).

SEM analysis illustrated the size and morphology of the samples. Figure 1 exhibited the SEM images of MCC and NCC. The initial evaluation of the pictures showed that MCC and NCC's morphological characteristics differed. The surface of MCC was smooth, while NCC exhibited a certain degree of fibrillation. The fibrillation may be attributed to sulfonate anion (–OSO_3_^−^) groups introduced on the cellulose composition. Despite the anionic charges on NCC fibrils inducing electrostatic repulsion among the microfibrils, the formation of hydrogen bonding between hydroxyl groups of the adjacent particles resulted in some agglomerations^22,23^.

#### EDX

The energy dispersive X-ray analysis (EDX) was also taken to determine the elemental composition of each sample (Fig. 1). The results represented the semi-quantitative vision of the element weight percentage in each composition. Whereas the detectable elements in MCC were carbon (C) and oxygen (O) with elemental rates of 50.10% and 49.90%, in NCC, carbon (C), oxygen (O), and sulfur (S) with amounts of 51.76, 46.98, and 1.26%, were detected, respectively.

#### TEM

**Figure 2:**
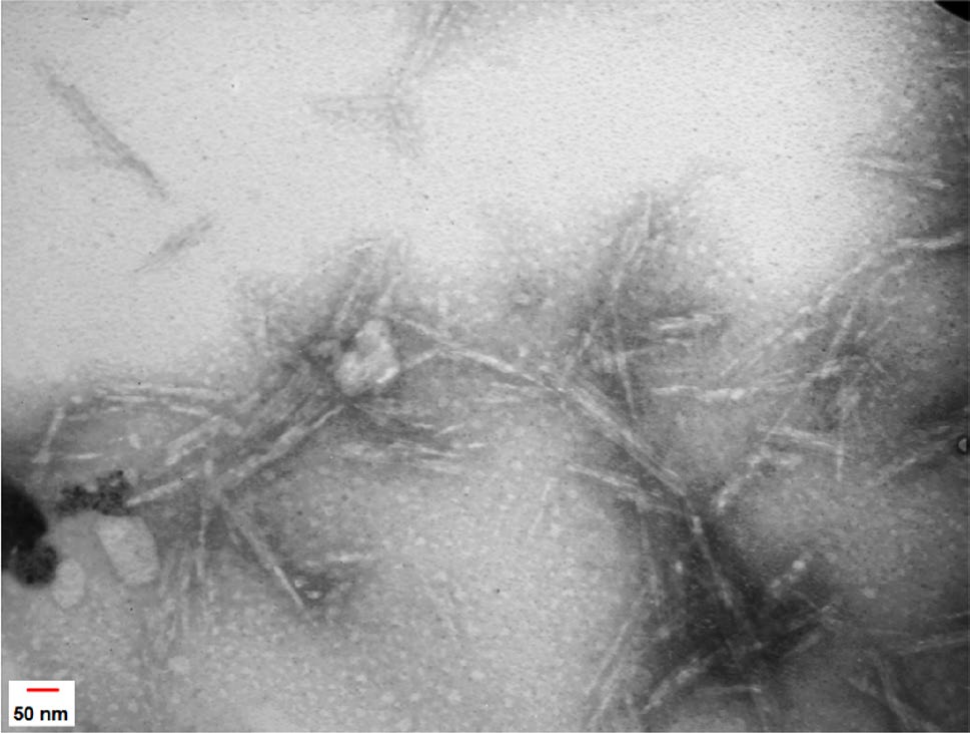
TEM analysis of NCC.

TEM was used to illustrate the morphology of the NCC produced by hydrolysis of MCC under strongly acidic condition (H_2_SO_4_; 65% v/v). However, previous studies have reported several morphologies for NCC according to the conditions and sources^24–26^. The prepared NCC exhibited a rod-and/or needle-like shape (Fig. 2). The TEM image demonstrated a length of 100–200 nm and a width of less than 10 nm for the NCC. Due to the sample preparation for the analysis (power of the sonication and NCC’s concentration), the distribution of the NCC was not homogenous in the TEM image.

#### XRD

**Figure 3:**
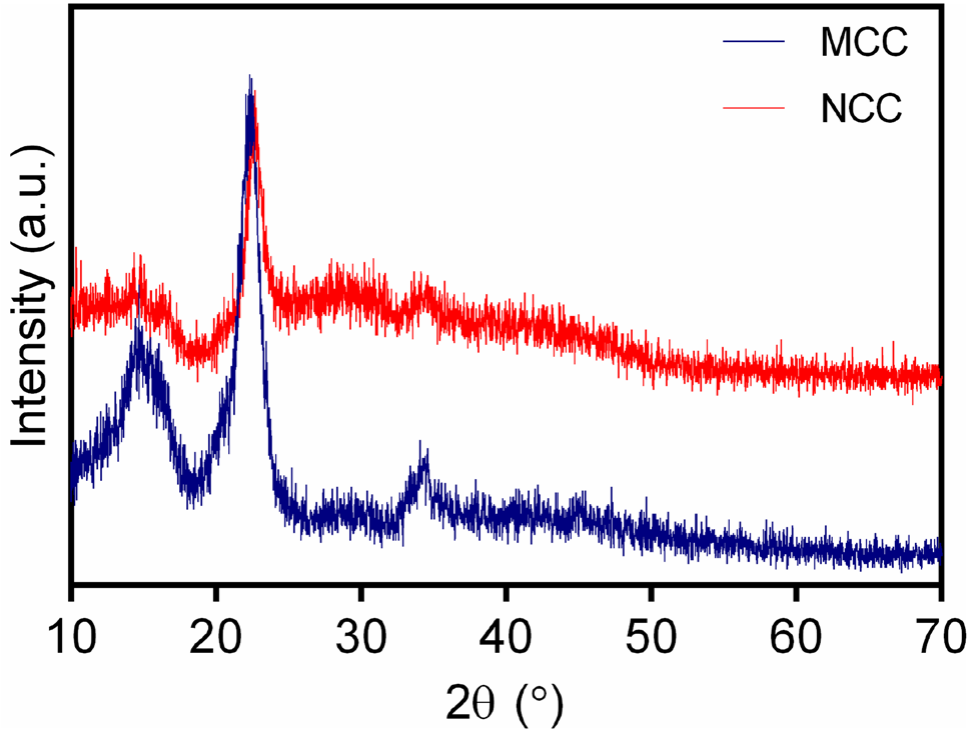
XRD patterns of MCC and NCC scanned over a range of 2θ = 10° to 70°.

XRD was utilized to exhibit the crystallinity of MCC and NCC. However, limited data could be obtained from the cellulose-based samples due to their low crystallinity^27^. As seen in Fig. 3, both MCC and NCC displayed broad and sharp peaks related to the amorphous and crystalline regions. NCC showed two broad peaks with low intensities around 14.4° and 34.52° and a sharp one at 22.84°. MCC illustrated three sharper and more intense peaks (15.2°, 22.68°, and 34.55°). All peaks correspond to the crystallographic planes that are (101), (002), and (040)^28^. The crystallinity index (CI) is a parameter to evaluate the relative amount of crystalline material^29^. The degree of crystallinity could be calculated using X-ray diffraction. One approach for X-ray diffraction analysis is using the Segal method due to the easiness of its application. The CI value is calculated using the Segal peak height method below^30^.

$$C{\text{I}}= \frac{{{\text{I}}}_{{\text{max}}}- {{\text{I}}}_{{\text{am}}}}{{{\text{I}}}_{{\text{max}}}} \times 100$$

I_max_ and I_am_ are attributed to the intensity of signals at around 2θ = 22.46° and 18.46°. The CI values calculated using the above equation for MCC and NCC were 51% and 56%, respectively. The CI of the synthesized NCC was observed to be higher than the MCC. Therefore, NCC had more crystalline sections than the MCC, which confirmed the removal of the amorphous portion during acidic hydrolysis.

#### DLS

**Figure 4:**
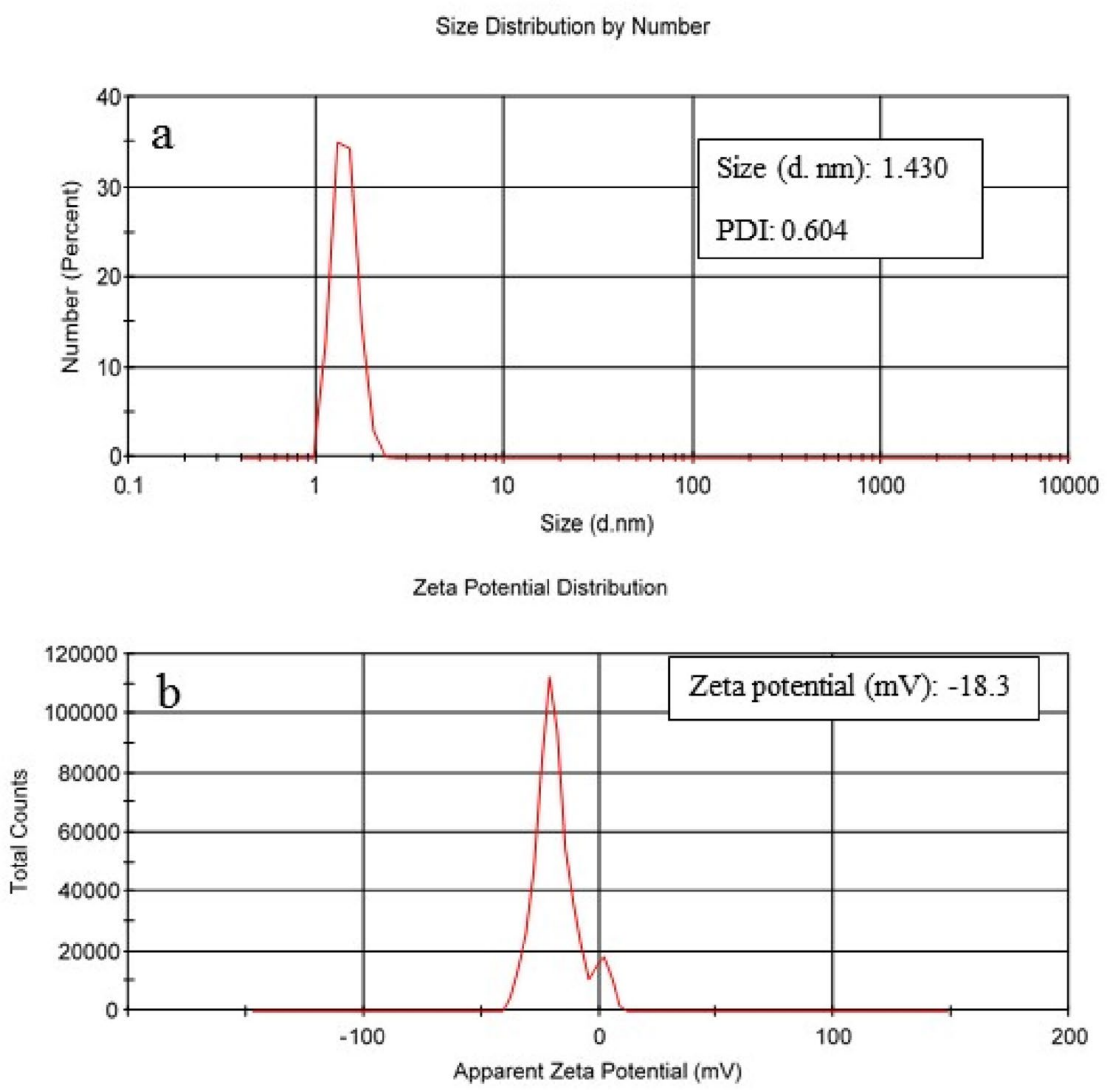
Particle size distribution (**a**) and zeta potential (**b**) of NCC.

DLS was carried out to estimate NCC's particle size distribution, polydispersity index, and zeta potential. According to Fig. 4a, the mean hydrodynamic diameter of the synthesized NCC was recorded as 1.430 ± 0.213 nm. The sharp peak and the recorded PDI value of 0.6 indicated a relatively uniform distribution. The surface charge was measured at − 18.3 mV, based on the zeta potential value (Fig. 4b), confirming the synthesized NCC's stability. In a study by A.B. Perumal et al., The SEM images of raw areca nut husk fibers indicated an uneven surface due to the cementing materials like pectin. Alkaline treatment followed by bleaching and sulfuric acid treatments eliminates cementing materials and amorphous sections. TEM images of obtained cellulose nanocrystals (CNCs) showed a rod-like structure with a length of 195 nm and a width of 19 nm. According to DLS results, the hydrodynamic size distribution was 578.2 nm, and the zeta potential was around − 15.3 mV. Moreover, a CI of 90% was reported from CNCs following XRD analysis. Using a different source may explain the varied results of this study^31^. To create a biocomposite packaging film, rice straw fibers were used to produce CNCs. The analysis revealed that CNCs with a needle-like shape were formed, with a length of 831 nm and a width of 44 nm. The average particle size was found to be 830.4 nm. The CNC surface has sulfate groups with negative charges, resulting in a zeta potential of − 30 mV. During the process, non-cellulosic compounds were removed, which led to an increase in the CI value from 42.85% for raw material to 71.37% for obtained nanocrystals^32^. CNCs derived from rice fibers were also used to develop bio-nanocomposite films based on chitosan and polyvinyl alcohol. SEM and TEM results indicated that the treatments removed amorphous regions, resulting in nanocrystals with a rod-like shape measuring 172 × 15 nm. The CNCs obtained had a zeta potential of − 10.4 mV and a CI value of 78%. Evaluation of conducted studies reveals that various parameters such as source and treatment method could alter the CNCs' characteristics^33^.

### The effects of MCC and NCC on growth parameters

**Figure 5:**
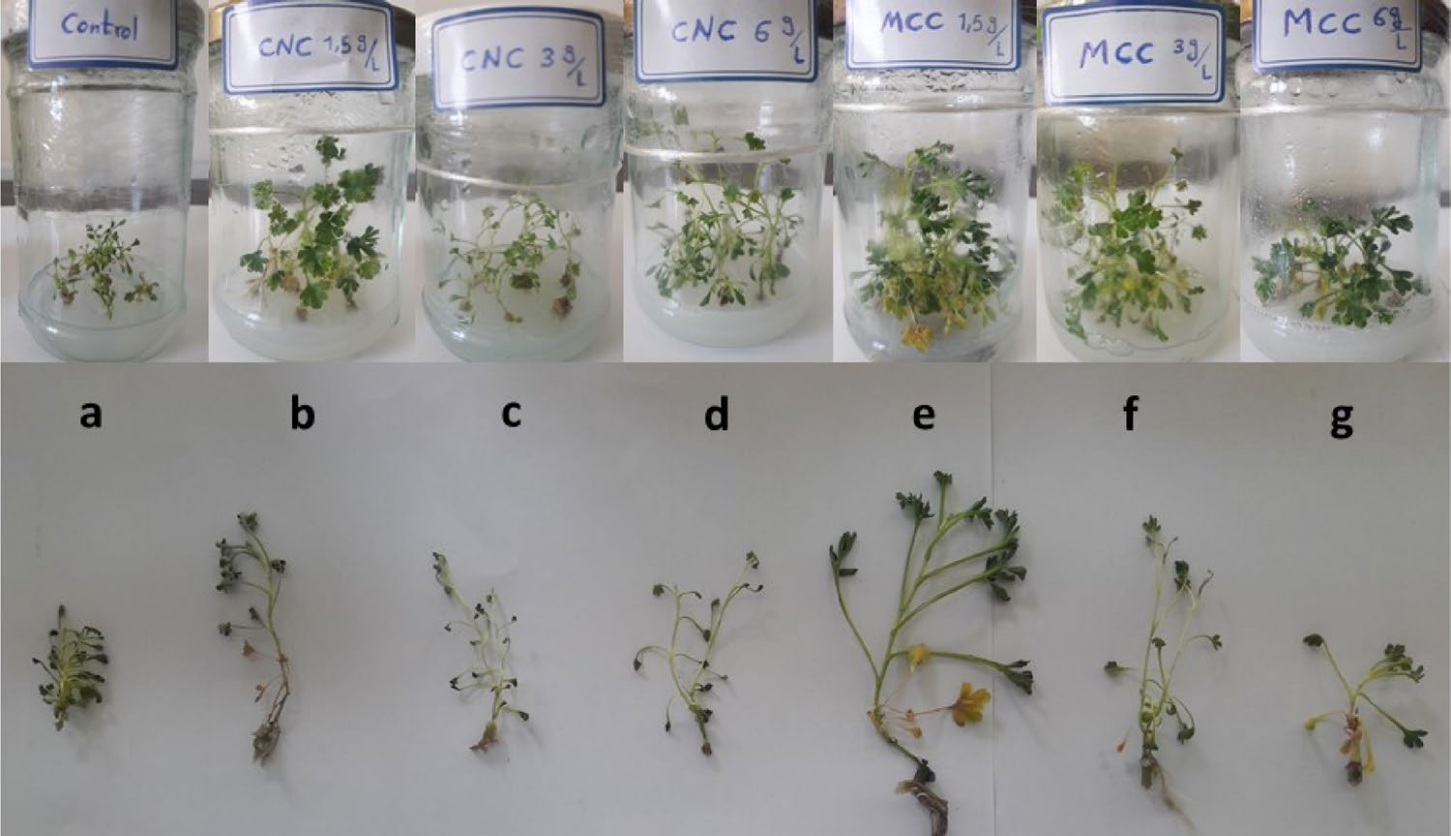
The effects of different MCC and NCC treatments on growth parameters of the grown shoots. (**a**) control, (**b**) 1.5 g/L NCC, (**c**) 3 g/L NCC, (**d**) 6 g/L NCC, (**e**) 1.5 g/L MCC, (**f**) 3 g/L MCC, (**g**) 6 g/L MCC.

**Figure 6:**
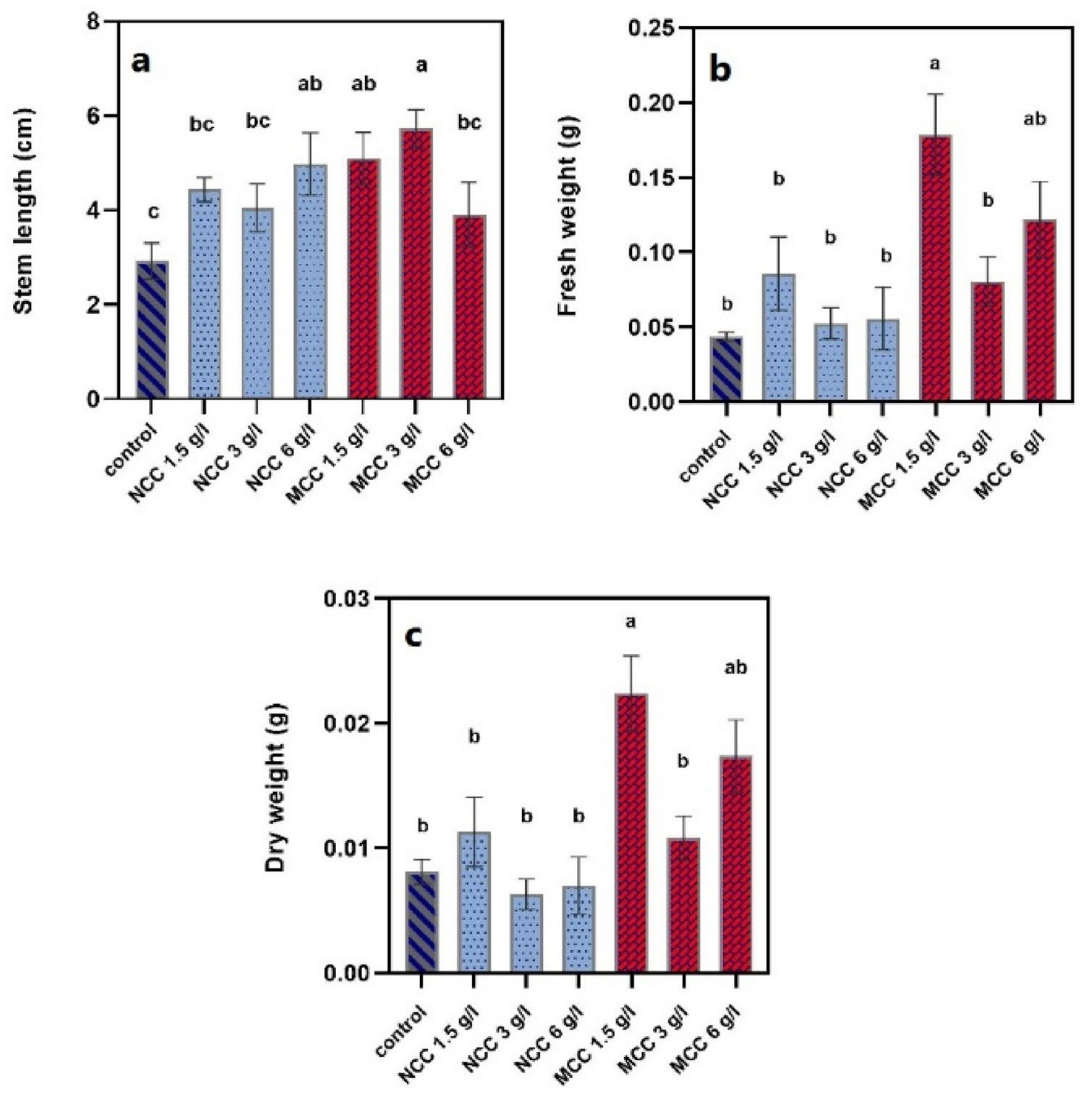
The effects of different MCC and NCC treatments on the stem length (**a**), fresh weight (**b**), and dry weight (**c**) of the grown shoots. Groups containing at least one common letter are not significantly different (*P* > 0.05).

Exposure to different concentrations of MCC and NCC did not significantly alter any of the growth parameters except for stem length, fresh and dry weights (Fig. 5). Treatments of 3 and 1.5 g/L MCC and 6 g/L NCC, in order of effectiveness, significantly increased the stem length of treated plants (*P* ≤ 0.05). Other treatments had little positive influence compared to the control group (*P* > 0.05). Regarding the fresh and dry weights, the significant increase (*P* ≤ 0.05) was only detected in plants treated with 1.5 g/L of MCC. Despite positively affecting the fresh weight, other treatments did not cause a statistically notable difference (*P* > 0.05). Besides, treatment with 3 and 6 g/l of NCC has decreased the dry weight insignificantly (*P* > 0.05) (Fig. 6). In line with these results, several studies have acknowledged the positive effects of polysaccharide nanoparticles on the growth parameters of some plants. Saharan et al.^34^ have reported that on maize; significantly higher values of all growth parameters, including shoot and seedling lengths along with fresh and dry weights, have been recorded at 0.04–0.12% concentrations of Cu-chitosan NPs. Similarly, in another study on maize (*Zea mays L*.), Cu-chitosan NPs, at 0.01–0.12% concentrations, showed a notable increase in plant height, stem diameter, root length, and root number^35^. Furthermore, 0.1%, 0.2%, and 0.3% of nano chitosan boosted the growth parameters (plant height, leaf area, fresh and dry weights of the shoot and root) of *Phaseolus vulgaris* significantly under salinity stress^36^. In another research, treating wheat plants with chitosan nanoparticles loaded with nitrogen, phosphorus, and potassium (NPK) substantially increased all growth variables (root and stem lengths, fresh and dry weights, water content, and leaf area)^37^. Another publication also demonstrated the striking growth-enhancing effects of Cu–chitosan nanoparticles on tomato seedling length, fresh and dry weights at 0.08, 0.10, and 0.12% levels^38^.

### Evaluation of photosynthetic pigments, total phenolic, and flavonoid contents, and anti-oxidant activity

**Figure 7:**
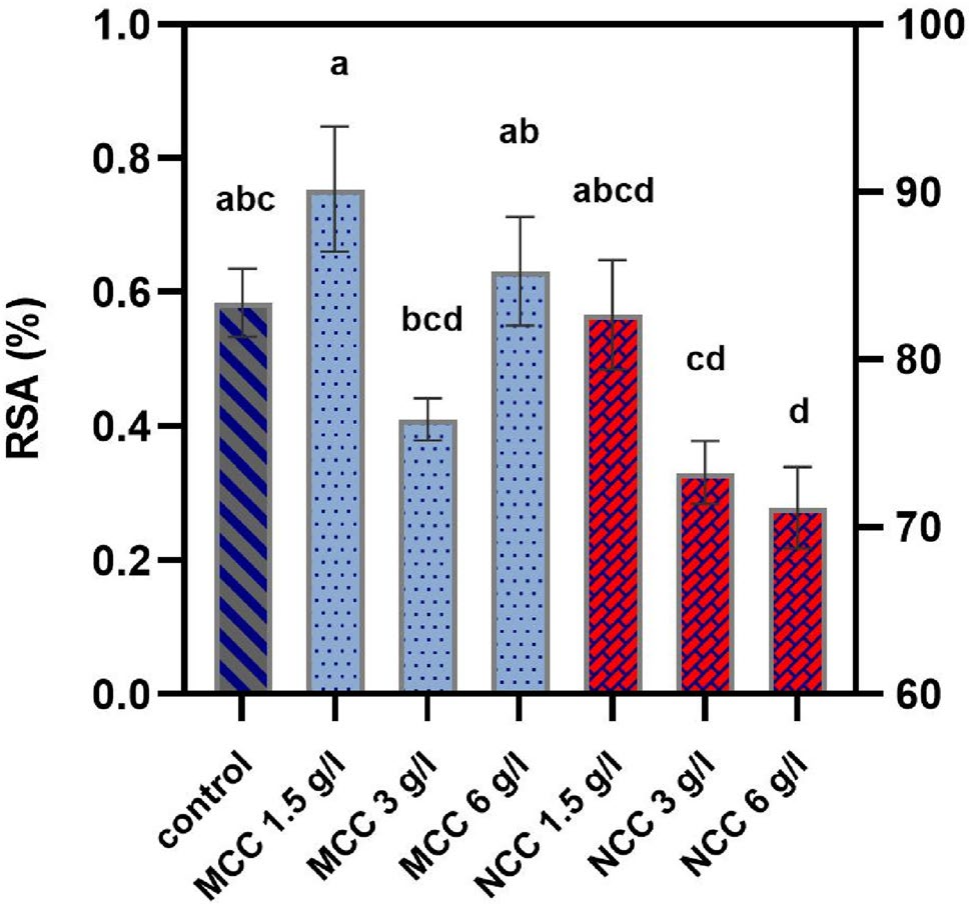
The effects of different MCC and NCC treatments on the radical scavenging activity of the grown shoots. Groups containing at least one common letter are not significantly different (*P* > 0.05).

The production of photosynthetic pigments, including chlorophyll a, b, and carotenoids in treated plants, was not changed significantly (*P* > 0.05). Likewise, the same result was obtained by comparing the total phenolic and flavonoid contents before and after treatment with MCC and NCC. Neither MCC nor NCC at the examined concentrations could have improved the above parameters (*P* > 0.05). Although no significant rise was observed in the phenolic content of different groups, the antioxidant activity of treated shoots demonstrated significant changes (*P* ≤ 0.05). A glance at Fig. 7 reveals that the highest antioxidant activity was observed in 1.5 g/L of MCC treatment, significantly higher than 3 g/L of MCC and 3 and 6 g/L of NCC treatments. On the other hand, 6 g/L of NCC had the lowest antioxidant activity, considerably less than the control and 1.5 and 6 g/L of NCC (*P* ≤ 0.05). In general, treatment with NCC had adverse effects and reduced the antioxidant activity compared to the control, which was a slight decrease in concentrations of 1.5 and 3 g/L (*P* > 0.05). Among MCC treatments, the concentration of 3 g/L slightly reduced the antioxidant activity (*P* > 0.05). However, this reduction was statistically significant compared to the 1.5 g/L of MCC treatment (*P* ≤ 0.05). Some studies, including Silva et al., have found that chitosan nanoparticles do not increase the total phenolic compounds in Sousão variety grapevine skins and stems but lead to a significant improvement in their antioxidant activity^39^. Additionally, two other studies have shown that chitosan treatment has no significant effect on the phenolic content of grapes and wine^40,41^. In contrast, 0.01 mg/mL of chitosan causes significant changes in total phenolic and flavonoid contents, besides the antioxidant activity of spinach leaves. In the same study, total chlorophyll content does not change, which is compatible with our observations^42^. Additionally, contrary to our findings, the chlorophyll a and b contents of maize (*Zea mays L*.) were elevated by Cu-chitosan NPs^35^. In mung beans under salinity stress, regular and nano-sized chitosan leads to higher total phenolics, flavonoids, chlorophyll a, b, and carotenoids^43^. Moreover, after chitosan elicitation, a greater level of the total phenolics and flavonoid accumulation of safflower callus under in vitro salinity stress was recorded^44^.

### The effects of MCC and NCC on volatile components

A total of 16 volatiles were identified by GC–MS analysis of in vitro-grown shoots of *A. absinthium* (Table 2). Treatments of 1.5 and 3 g/L of MCC, containing 15 and 7 components, respectively, had the greatest and the smallest number of volatile compounds. The control had the second-largest number of detected volatile compounds (13 components). The main constituents of volatiles were 1-Hexene and, 1-Pentene, 2-methyl. The majority of compounds in all treatments were non-terpenoids, consisting mainly of alkene and alkane hydrocarbons. Only two monoterpenes and one diterpene were found.

**Table 2:** Composition of volatile compounds from the shoot culture of *A. absinthium* under MCC and NCC treatments.

No.	Constituents	Peak area (%)								RT	RI	References
		Control	MCC 1.5 g/L	MCC 3 g/L	MCC 6 g/L	NCC 1.5 g/L	NCC 3 g/L	NCC 6 g/L	Similarity %			
1	1-Hexene	34.8	27.29	33.65	67.6	17.6	26.4	73.04	90	5.1	584	^45^
2	1-Pentene, 2-methyl	40.59	30.46	34.02	–	19.96	30.57	–	90	5.13	588	^46^
3	Cyclopentane,1,3-dimethyl	6.86	5.97	9.12	2.15	4.61	7.51	9.9	95	5.306	682	^47^
4	1,2-Propanediol	0.88	14.4	0.36	11.8	7.3	2.06	3.82	98	5.575	710	^48^
5	2-Hexanone	1.2	1.06	–	0.45	0.4	–	1.16	96	6.311	768	^49^
6	Heptane, 2,4-dimethyl	0.31	–	–	–	–	–	–	94	6.791	825	^49^
7	β-Thujone	0.63	0.73	–	–	–	–	–	95	12.936	1081	^50^
8	2,4-Di-tert-butyl phenol	1.07	0.92	0.3	0.8	0.46	0.51	1.16	91	20.326	1435	^51^
9	Hexadecane	4.4	0.46	–	0.75	–	–	–	94	20.495	1558	^52^
10	Geranyl isovalerate	–	0.41	–	–	–	–	0.8	92	21.499	1590	^53^
11	Nonadecane	0.38	0.32	–	–	–	–	0.4	93	23.944	1890	^52^
12	Palmitic acid	1.32	0.92	0.42	1.35	0.71	0.77	1.3	92	27.025	1981	^54^
13	Linoleic acid	–	0.73	–	1.2	0.75	0.82	–	90	29.233	2130	^55^
14	Phytol	–	0.32	–	–	–	–	–	91	29.24	2138	^55^
15	Oleic acid	2.77	2.44	1.21	3.01	2.43	2.73	1.46	91	29.326	2152	^55^
16	Stearic acid	1.89	1.89	–	1.25	–	1.34	–	86	29.594	2187	^55^
	Total	97.1	88.32	79.08	90.36	54.22	72.71	93.04				
	Terpenoids	0.63	1.46	–	–	–	–	0.8				
	Non-terpenoids	96.47	86.86	79.08	90.36	54.22	72.71	92.24				

RT: Retention time, RI: Retention indices were collected from NIST Chemistry WebBook and previous articles for the DB-1 column.

β-thujone was only detected in control and 1.5 g/L of MCC. Therefore, it seems likely that other treatments inhibited the production of this component. Linoleic acid, phytol, and geranyl isovalerate were some compounds not found in control but were produced and detected under some MCC and NCC treatments. Phytol and geranyl isovalerate production was only boosted by 1.5 g/L of MCC, and linoleic acid was induced under the influence of 1.5 and 6 g/L of MCC and 1.5 and 3 g/L of NCC. Other fatty acids found in control, such as oleic acid, palmitic acid, and stearic acid, were found in other treatments in approximately similar amounts.

Plants' essential oils possess significant biological activities, including antifungal effects. For instance, the essential oil of green tea demonstrates antifungal activity against *Magnaporthe oryzae *in vitro. Other essential oils, such as anise, thyme, clove, and cinnamon, also inhibit the growth of some plant tissue fungal strains. Therefore, it is important to evaluate the content of volatile oils to identify active components and increase their production yield^56–58^. The study of volatile compounds of *A. absinthium*, grown in different conditions and habitats, has indicated different results. In a survey on wormwood grown in Iran, it was found that about half of the volatile compounds are hydrocarbon monoterpenes, and β-pinene, β-thujone, sabinene, myrcene, and linalool are the main components^59^. Additionally, myrcene, β-thujone, cis-chrysanthenyl acetate, and linalool have been reported as the major volatile compounds of wormwood collected from Tajikistan^60^. Some European plants (France, Croatia, and Romania) were examined. The major volatile constituents of the wormwood collected from France included (Z)-epoxyocimene and chrysanthenyl acetate, while in wormwood collected from Croatia, (Z)-epoxyocimene and β-thujone were the major components^61^. A significant number of monoterpenes were identified in Romanian wormwood, which were mainly hydrocarbons. The most abundant ones were β-pinene, α-pinene, pseudolimonen, geranyl bromide, and terpinolene^62^. However, our study found only tiny amounts of β-thujone and none of the other compounds. This suggests that the volatile compound composition of this plant is highly diverse within the same species, and several factors can affect essential oil content. It seems essential oil content depends on environmental factors and growth conditions, habitat, plant chemotype, and even the extraction method. Meanwhile, a study compared the essential oil content of *A. absinthium* grown in vitro, greenhouses, and fields. The study found that seedlings grown in vitro did not generally contain monoterpenes, which agrees with our results. The major volatile compounds in the in vitro sample were citronelyl isovalerate and terpinyl isobutyrate, neither found in plants grown in greenhouse and field, and the main content of greenhouse and field plants was α-thujone^63^. Bicyclic monoterpenes, specifically thujones, are the most significant constituents of wormwood essential oil. Other important components that have been reported include myrcene, sabinene, linalool, cis-epoxyocimene, chrysanthenyl acetate, and trans-sabinyl acetate. However, in the present study, which was conducted in vitro, notable differences are observed in the main chemical compounds compared to in vivo studies.

### The effects of MCC and NCC on the production of phenolic acids

The in vitro-grown shoots were examined in terms of the phenolic acids content, specifically for caffeic acid (Rt = 2.80 min), salicylic acid (Rt = 3 min), vanillic acid (Rt = 3.02 min), *p*-coumaric acid (Rt = 3.61 min), ferulic acid (Rt = 3.63 min), *m*-coumaric acid (Rt = 4.24 min), rosmarinic acid (Rt = 4.49 min), cinnamic acid (Rt = 5.24 min) and gallic acid (Rt = 5.55 min). Based on the data presented in Table 3, it is evident that in the control group, only gallic, caffeic, and p-coumaric acids were detected as phenolic acids. However, some treatments led to ferulic acid and salicylic acid synthesis. In particular, ferulic acid production was stimulated by the 3 g/L and 6 g/L MCC treatments, while salicylic acid synthesis was induced by all NCC treatments and the 3 g/L MCC treatment. The level of gallic acid was improved significantly in all samples except for 1.5 g/L of MCC. The amount of *p*-coumaric acid has been boosted in all treatments but was not detected in 1.5 and 6 g/L of NCC. The caffeic acid contents have also declined in all treatments. The enhancement of gallic acid levels in 6 g/L of NCC and 3 g/L of MCC treatments and *p*-coumaric and salicylic acids in 3 g/L of NCC were more considerable than in the other treatments. Also, the synthesis of phenolic acids (such as *p*-coumaric, caffeic, ferulic, and gallic acids, which have antimicrobial activity) in wheat primary leaves treated with chitosan ameliorates significantly with increasing chitosan concentration^64^. Chitosan treatments enhance the acid accumulation up to 10.3-fold in cell suspension cultures of Malus X Domestica Borkh (apple)^65^. The previous in vivo studies regarding *A. absinthium* phenolic acids content confirmed the presence of all acids examined in the current study^66–68^. However, in contrast to these studies, our in vitro experiment did not detect vanillic, m-coumaric, rosmarinic, and cinnamic acids in any of the treatments. This could be due to the different vegetative stages of the plants used.

**Table 3:** Phenolic acids content of the grown shoots of *A. absinthium* under different MCC and NCC treatments (mg/g d.w.).

Treatments (g/L)	GA	CAA	FA	*m*-COU	*p*-COU	CA	RA	SA	VA
Control	2.77	2.21	–	–	0.002	–	–	–	–
MCC 1.5	2.54	1.81	–	–	0.005	–	–	–	–
MCC 3	9.97	1.85	0.057	–	0.005	–	–	0.001	–
MCC 6	3.42	1.70	0.009	–	0.005	–	–	–	–
NCC 1.5	3.70	0.93	–	–	–	–	–	0.003	–
NCC 3	3.10	0.76	-	–	0.010	–	–	0.015	–
NCC 6	12.0	1.94	–	–	–	–	–	0.001	–

The sign (–) indicates that the substance was not found in the treatment. (GA: gallic acid, CAA: caffeic acid, FA: ferulic acid, *m*-COU: meta coumaric acid, *p*-COU: para coumaric acid, CA: cinnamic acid, RA: rosmarinic acid, SA: salicylic acid, VA: vanillic acid).

### Morphological observations of treated shoots using SEM

**Figure 8:**
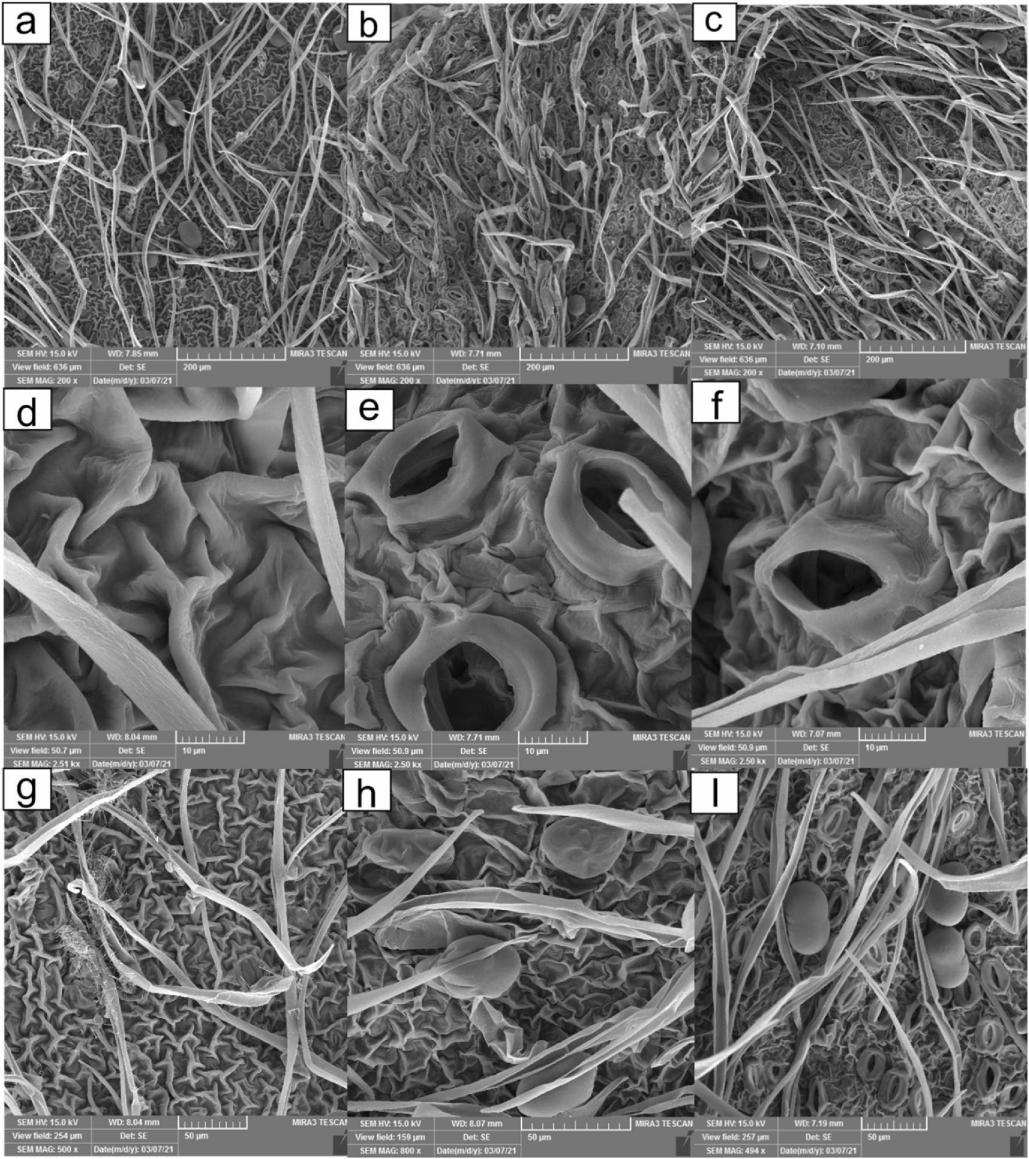
SEM observations were conducted on the surface of leaves from control plants (**a**, **d**, **g**), plants treated with 1.5 g/L of MCC (**b**, **e**, **h**), and plants treated with 1.5 g/L of NCC (**c**, **f**, **i**). The observations included leaf hairs from the control plants (**a**), leaf hairs from the plants treated with 1.5 g/L of MCC (**b**), leaf hairs from the plants treated with 1.5 g/L of NCC (**c**), stomata from the control plants (**d**), stomata from the plants treated with 1.5 g/L of MCC (**e**), stomata from the plants treated with 1.5 g/L of NCC (**f**), glandular trichomes from the control plants (**g**), glandular trichomes from the plants treated with 1.5 g/L of MCC (**h**), and glandular trichomes from the plants treated with 1.5 g/L of NCC (**i**).

Figure 8 provided the morphological changes of the leaves under the effects of 1.5 g/L of MCC (b, e, h) and NCC (c, f, i) contrasted to the control (a, d, g). It was apparent from the images supplied that all three groups contained leaf hairs and did not differ considerably in the frequency of hairs (a-c). No stomata were observed in the control group, probably due to their low frequencies in control (a, d, g). In contrast, stomata were vividly visible in the MCC and NCC-treated leaves, and their frequency had significantly increased (b, c, e, f). In addition, the number of glandular trichomes in treated plants has increased relatively (a-c, g-i). The increase in the number of stomata and glandular trichomes could be explained by the enhancement of some growth parameters in the presence of MCC and NCC, as well as the increase in the production of volatile compounds by exposure to 1.5 g/L of MCC. SEM images also revealed the above correlation. In agreement with our results, it has been previously reported that applying chitosan nanoparticles in agriculture could increase the number of stomata and stomatal conductance^69^. A study conducted on Dendrobium Orchid reported a positive effect of chitosan on the number of stomata at concentrations of 10, 20, 40, and 60 mg/L^70^. Similarly, using chitosan on soybean plants has also increased stomata density^71^. However, fewer but larger stomata were found when different concentrations of chitosan were used on the adaxial leaf side of the Dendrobium hybrid^72^.

## Conclusion

This study reports the first use of MCC and NCC in plant culture media. The study found that the lowest concentration of MCC was the most effective at improving the production of secondary metabolites and growth parameters of *A. absinthium*. Some concentrations of MCC also increased antioxidant activity and growth parameters. Regarding volatile compounds, non-terpenoids were the most prevalent in all treatments. Some concentrations of both MCC and NCC induced the production of ferulic and salicylic acids, which were not detected in the control. Additionally, the lowest concentration of both MCC and NCC increased the number of stomata and glandular trichomes.

### Supplementary Information

Supplementary Information.

## Data Availability

The datasets used and analyzed during the current study are available from the corresponding author upon reasonable request.
