# Supplementary figures and images for: The impact of microcrystalline and nanocrystalline cellulose on the antioxidant phenolic compounds level of the cultured Artemisia absinthium
Source: Sci Rep. 2024 Feb 1;14:2692. doi: 10.1038/s41598-023-50772-3 (PMC10834404; doi:10.1038/s41598-023-50772-3)

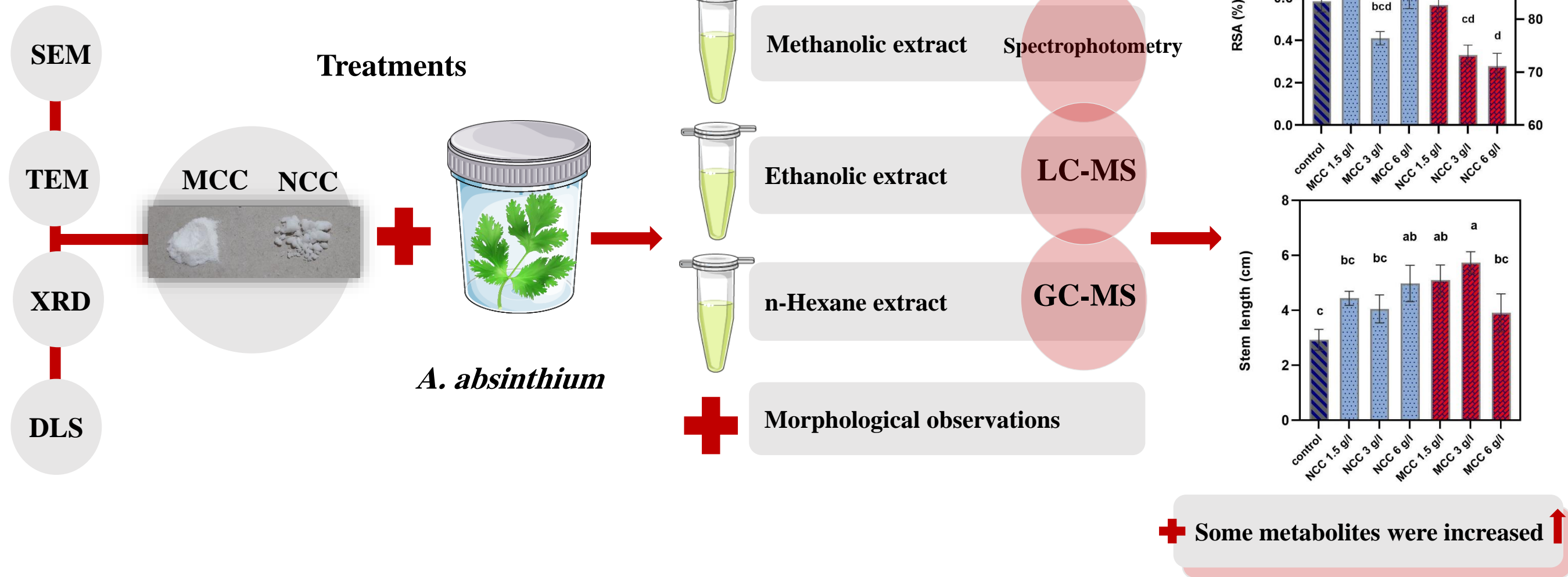

Supplement: Supplementary file 1 — Supplementary Information. [file 41598_2023_50772_MOESM1_ESM.pdf]
